# Prediction of Chemical Composition and Sensory Information of *Codonopsis Radix* Based on Electronic Nose

**DOI:** 10.3390/molecules30051146

**Published:** 2025-03-03

**Authors:** Xingyu Guo, Ruiqi Yang, Yushi Wang, Jiayu Wang, Yashun Wang, Huiqin Zou, Yonghong Yan

**Affiliations:** School of Chinese Materia Medica, Beijing University of Chinese Medicine, Beijing 102401, China; 20230941454@bucm.edu.cn (X.G.); yangrq@bucm.edu.cn (R.Y.); 20230935206@bucm.edu.cn (Y.W.); 20230935245@bucm.edu.cn (J.W.); 20200941404@bucm.edu.cn (Y.W.)

**Keywords:** *Codonopsis Radix*, electronic nose, SVM, regression prediction

## Abstract

*Codonopsis Radix* (CR), an important species of “medicine-food homology”, exhibits broad market prospects, underscoring the urgency and importance of research on its quality. This study specifically measured the alcohol-soluble extract and polysaccharide extract of 77 samples from mainstream producing areas of CR, which serve as key fractions for assessing its quality. Additionally, to gain a comprehensive understanding of the sensory characteristics of samples, the study employed electronic tongue technology to obtain sweetness values, used a colorimeter to determine yellowness values, and captured odor fingerprint information through an electronic nose (E-nose). In the data analysis phase, the study compared the accuracy of various regression prediction models, including Multiple Linear Regression (MLR), Random Forest (RF), Support Vector Machine (SVM), and Artificial Neural Network (ANN). After comprehensive evaluation, an SVM algorithm was selected due to its superior prediction performance. To further enhance prediction accuracy, the study utilized a Particle Swarm Optimization (PSO) algorithm to optimize the SVM, resulting in a significant improvement in the prediction accuracy of sweetness values. In conclusion, regression prediction models for chemical composition and sensory information of CR based on an E-nose were established. It represents an enhancement of traditional morphological identification methods for Chinese medicinal herbs and provides new ideas and means for quality evaluation of CR. Furthermore, it offers a reference for quality evaluation of other similar Chinese medicinal herbs.

## 1. Introduction

CR, a medicinal plant with tonic effects, can also be used as food [1]. The types of chemical components in CR include flavonoids, alkynes, alkaloids, phenylpropanoids, lignans, steroids, triterpenes and saponins, polysaccharides, etc. [2]. In addition, it is also rich in essential trace elements such as iron, silicon, aluminum, selenium, manganese, etc. [3]. When used as a traditional Chinese medicine, CR has the effects of strengthening the spleen, benefiting the lungs, nourishing blood, and generating fluids [4]. When used as food, CR can be made into medicinal meals and health care products [5], which can enhance immunity, improve anemia, and strengthen physique, and is beneficial to people’s health [6].

CR is a multi-source traditional Chinese medicine. *Chinese Pharmacopoeia* (2020 edition) stipulates that the original plants of CR include *Codonopsis pilosula* (Franch.) Nannf., *Codonopsis pilosula* Nannf. var. *modesta* (Nannf.) L.T.Shen, and *Codonopsis tangshen* Oliv. Due to factors such as the climate environment, cultivation methods, harvesting time, and processing methods, there are differences in the quality of different *Codonopsis pilosula* samples [7]. For example, with the increase in growth years, the immunoreactivity, antioxidation, and comprehensive scores of efficacy components and nutrients of CR showed an increasing trend [8]. The results of fingerprinting combined with chemometric methods showed that the quality of CR varied among different origins [9]. Similar results were obtained by histological techniques combined with network pharmacological analysis [10].

Currently, the quality evaluation methods of CR mainly focus on the determination of the content of chemical components [11], but no conclusions have been reached. *Chinese Pharmacopoeia* (2020 edition) only specifies the content of alcohol-soluble extracts [12]. Additionally, polysaccharide is the most popular. It has activity in neuroprotection [13], hepatoprotection [14], glucose regulation [15], antitumor [16], etc., and the Gansu Provincial Health Commission has supplemented polysaccharide in the local standard (DBS62 001-2021). E-nose is an intelligent biomimetic technology consisting of a sampling system, a sensor system, and a signal processing system, which simulates human smell. With powerful sensor systems and machine learning algorithms, it can recognize subtle differences between different samples [17]. In the early stage, we found that the varieties of CR can be well identified by odor information, as well as samples from different origins of the same variety. Thus, is there a correlation between the odor of CR and its internal components? Is there a correlation between the odor of CR and other external sensory information such as color and taste?

In conclusion, this study determined the recognized index components and sensory information of different CRs and explored the feasibility of using E-nose to achieve rapid, simple and accurate detection of information from CR powder with correlation analysis and machine learning algorithms, which were a supplement to the quality evaluation methods of CR.

## 2. Results and Discussion

### 2.1. Chemical Composition and Sensory Test Results

**Figure 1:**
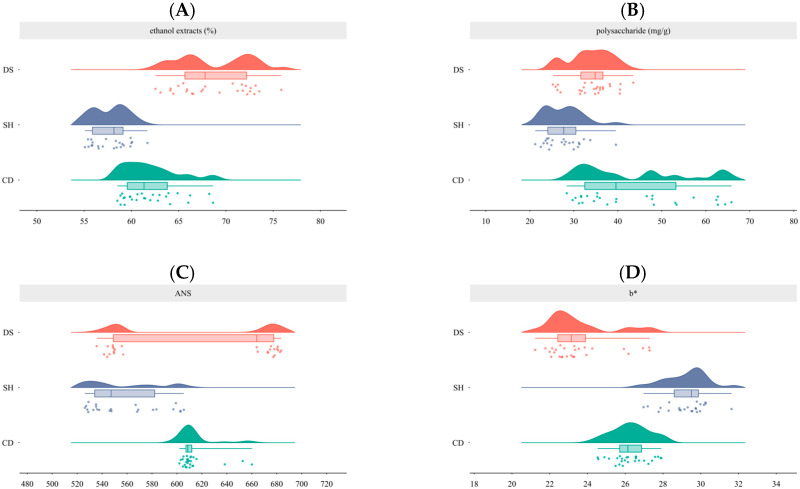
Cloud and rain maps. (**A**) Alcohol-soluble extract (%). (**B**) Polysaccharide (mg/g). (**C**) ANS values. (**D**) b* values. DS stands for *Codonopsis pilosula* (Franch.) Nannf.; SH stands for *Codonopsis pilosula* Nannf. var. *modesta* (Nannf.) L.T.Shen; CD stands for *Codonopsis tangshen* Oliv.

According to the above methods, all samples were tested and the results were visualized by cloud and rain maps. Figure 1A shows the results of the alcohol-soluble extract content, and all samples met the requirements specified in *Chinese Pharmacopoeia* (2020 edition). For the determination of the polysaccharide extract, the linear relationship OD value (y) versus concentration (x, mg/mL) conformed to y = 0.1816x + 0.0001 with *R*^2^ = 0.9994, and the concentration ranged from 0.0069 to 0.1100 mg/mL. Methodological results showed that the RSD value of precision test was 0.64%, the reproducibility test was 2.53%, the stability test within 24 h was 1.02%, the spiked recoveries test was 1.49%, and the spiked recoveries were in the range of 98.65%~102.24%, which passed the methodological test. Figure 1B shows the results of the polysaccharide extract determination.

*Chinese Pharmacopoeia* (2020 edition) describes the characteristic of CR as having a slightly sweet taste. Modern research has shown that the main sources of sweetness in CR were fructose and sucrose, which account for over 90% of free sugar components [18]. An electronic tongue was used to obtain objective values for the sweetness of CR, and the results are shown in Figure 1C. In addition, CR is yellowish in color. A colorimeter was used to obtain objective values for the yellowness of CR, and the results are shown in Figure 1D.

The above results indicate that there were differences in the alcohol-soluble extract content, polysaccharide extract, sweetness, and color between different CR samples. Among them, the values of the alcohol-soluble extract content, polysaccharide extract, and color were relatively concentrated, while the sweetness value varied greatly in samples with *Codonopsis pilosula* (Franch.) Nannf. as the original plant, clearly divided into two categories. Based on analysis of the sample sources, the reasons may be related to geographical origin factors, in which the samples with smaller sweetness values were all from Gansu, while the samples with larger sweetness values were all from Shanxi. This study did not involve an analysis of the quality difference of CR; therefore, the above issue was not the focus of attention. So, in the face of unknown powder samples, is it possible to use a method that can quickly and accurately predict all four indicators at the same time? Next, we used the E-nose to obtain odor information of CR by rich data results and combined these with machine learning algorithms to attempt to regressively predict the above four indicators.

### 2.2. E-Nose Analysis

**Figure 2:**
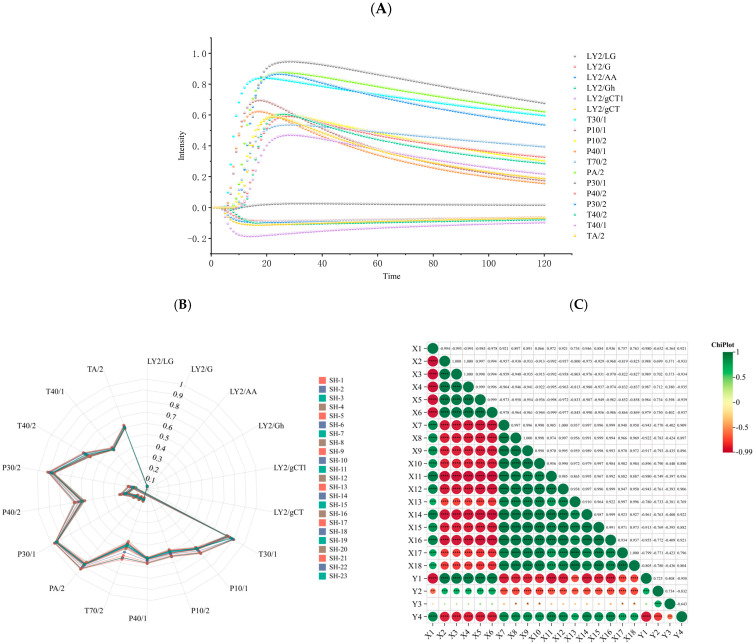
Results of E-nose analysis. (**A**) Odor fingerprint information. (**B**) Radar chart. (**C**) Correlation analysis (* <0.05; ** <0.01; *** <0.001; **** <0.0001).

Refer to the above method to obtain odor information for all samples. Taking SH-1 sample as an example, display odor fingerprint information, as shown in Figure 2A. Taking *Codonopsis pilosula* Nannf. var. *modesta* (Nannf.) L.T.Shen as an example, display the radar chart, as shown in Figure 2B. It could be seen that there were differences in odor in the different samples, which had potential for regression prediction of sample information. Correlation analysis was conducted among maximum response values of 18 sensors and alcohol-soluble extract content, polysaccharide extract, ANS response values, as well as b* values, as shown in Figure 2C. In the figure, X1~X18 represented 18 sensors, while Y1~Y4 represented the alcohol-soluble extract content, polysaccharide extract, ANS values, and b* values in sequence. The larger the spot was, the stronger was the correlation; the greener the spot color was, the positive correlation, the redder the spot color was, the negative correlation. The results demonstrate a correlation between the ANS value, reflecting the sweetness of CR, and several sensors of the E-nose. Additionally, there was a strong correlation observed between values of the alcohol-soluble extract, polysaccharide, and b* parameter (indicating yellow coloration of CR) and maximum response values recorded by all 18 sensors of the E-nose. Based on this odor fingerprint information, it is feasible to further develop a predictive model for the chemical composition and sensory characteristics of CR.

### 2.3. Constructing Regression Prediction Models Based on Machine Learning Algorithms

MLR is a linear model that derives the line of best fit by least squares and other algorithms to minimize the perpendicular distance from each data point to the line of fit, and is a supervised learning model that can be used for prediction [19]. RF belongs to the tree structure, optimizing model by integrating algorithms, which has advantages of easy interpretation and wide application [20]. SVM is the model closest to deep learning, most popular before the emergence of ANN, applicable to a case of small sample size [21]. ANN theory comes from human brain neurons, and is a complex adaptive nonlinear dynamic system composed of simple neuron basic elements. It can predict specific results at high granularity through data mining, and can find patterns and relationships in the data, which can better identify information with strong similarity [22]. Therefore, we first took the alcohol-soluble extract as an example to select the most suitable algorithm for constructing a regression prediction model, and then complete the prediction of other indicators.

#### 2.3.1. Select the Optimal Algorithm

We constructed an MLR prediction model for alcohol-soluble extract content of CR based on odor information using SPSS 20 software. Correlation results of 77 batches of samples showed that the Durbin–Watson test result was 1.709, around 2, indicating a weak autocorrelation and independence of samples, and regression analysis can be used. The maximum response value of the 9th sensor, T70/2, was significantly correlated with the content of the alcohol-soluble extract, with statistical significance (*p* < 0.01), while the rest had no significant correlation and were excluded variables. We randomly selected 70% of samples as a training set to establish the MLR prediction model; the regression equation obtained was Y = −69.934X_9_ + 103.523, the remaining 30% of samples was used as a testing set, the maximum response value of the 9th sensor was brought into the equation to obtain the predicted value of the alcohol-soluble extract content, while bivariate correlation analysis was conducted with the measured value, and the results show that the correlation coefficient was 0.755, *p* < 0.01, and the model was validated. However, since the absolute value of the correlation coefficient of MLR is closer to 1, this indicates that variables have a stronger linear relationship. Therefore, more samples were needed to verify the accuracy of the model.

We constructed regression prediction models for the alcohol-soluble extract content of CR based on odor information using MATLAB R2023b software, with 70% of samples as a training set and 30% of the samples as a testing set. In RF, the number of decision trees was set to 80, and the minimum number of leaves was five; in SVM, the penalty factor was set to three, and the radial basis function parameter was 0.8; in the Back Propagation Neural Network (BPNN), the number of neurons was set to 12, the number of iterations was set to 1000, the error threshold was set to 10^−6^, and the learning rate was set to 0.01. The predictive performance indices of the above models are shown in Table 1. Among them, RMSE is the root mean square error, which measures the difference between predicted and true values. The smaller the value is, the smaller the error and the better the model performance are; *R*^2^ is the coefficient of determination, which evaluates the fit of the model. The closer it is to 1, the better the fit and the better the model performance are; MAE is the mean absolute error, and the smaller the value is, the more accurate is the model prediction. Comprehensively evaluating the indicators of the model prediction performance, it was considered that SVM performs better among the above four models. Therefore, SVM was selected for prediction of subsequent indicators.

#### 2.3.2. Prediction of Composition and Sensory Information Based on SVM

The code was obtained from GitHub (https://github.com/Time9Y/Matlab-Machine, accessed on 28 October 2024), and parameters were set as in Section 3.2.1. Regression predictions were made for alcohol-soluble extract content, polysaccharide extract, sweetness values, and yellow values, and the results are shown in Table 2. It can be seen that the combination of odor information with SVM was feasible for predicting the above four indicators of CR, and performs well in predicting alcohol-soluble extract, polysaccharide, and yellow values, with determination coefficients *R*^2^ above 85%. However, taste information combined with the support vector machine model performed poorly in the prediction of sweetness values. Based on the correlation analysis results in Section 3.2, it was found that the maximum response values of 18 sensors have low correlation with sweetness values. Thus, could we consider optimizing the algorithm to improve the prediction performance? Next, we used PSO algorithm to optimize the SVM to re-predict the sweetness values [23].

**Figure 3:**
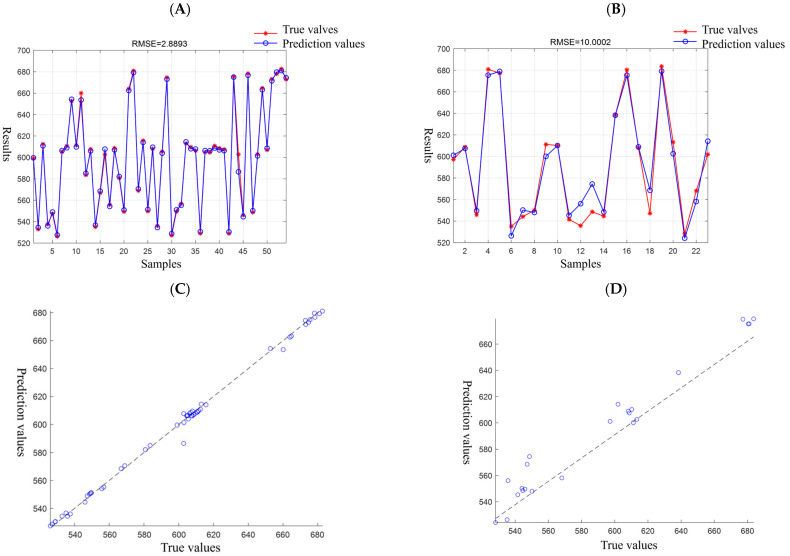
PSO-SVM results. (**A**) Comparison of training set prediction results. (**B**) Comparison of test set prediction results. (**C**) Prediction values of training set vs. true values of training set. (**D**) Prediction values of test set vs. true values of test set.

PSO-SVM results are shown in Figure 3; optimization occurred 100 times, each time with 10 particles; the training set to do fivefold cross-validation, to cross-validation of the root mean square error as the fitness value, the penalty factor, radial basis function parameters adjustment range were set to 0.1–100; the best values obtained were 18.4791 and 2.9800, respectively. *R*^2^ values for prediction performance indicators of training and testing sets were 0.996 and 0.962, and MAE values were 1.936 and 7.257. The model had better performance.

## 3. Materials and Methods

### 3.1. Samples and Instruments

#### 3.1.1. Samples

A total of 14 batches of CR from Changzhi, Shanxi, 13 batches from Dingxi, Gansu, 8 batches from Longnan, Gansu, 15 batches from Aba, Sichuan, 15 batches from Wushan, Chongqing, and 12 batches from Enshi, Hubei, were collected. All of them were harvested in the fall/winter of 2023, with a total of 77 batches of samples, as shown in Table 3. All samples were purchased from their place of origin and dried according to local experience and customs in processing CR medicinal materials, resulting in products that can be sold directly. And it was identified by Professor Yonghong Yan of the Beijing University of Traditional Chinese Medicine.

#### 3.1.2. Instruments

A FW-100 high-speed universal pulverizer model (Beijing Kewei Yongxing Instrument Co., Ltd., Beijing, China); an AK20002 electronic balance (Chengdu Besec Instrument Research Institute, Chengdu, China); KQ-500DE CNC Ultrasonic Cleaning (Kunshan Ultrasonic Instrument Co., Ltd., Kunshan, China); an HH-S6A electric constant temperature water bath (Beijing Kewei Yongxing Instrument Co., Ltd., Beijing, China); a drying oven (Shanghai Yiheng Scientific Instrument Co., Ltd., Shanghai, China); Multiskan FC Microplate Reader (Thermo Fisher Shanghai Instruments Co., Ltd., Shanghai, China); a GTR16-2 Pharmaceutical Centrifuge (Beijing Times Beili Centrifuge Co., Ltd., Beijing, China); a 20 mL headspace injection bottle; CM-5 spectrophotometer (Konida Minolta, Chiyoda City, Japan); a liquid taste analyzer (Alpha M.O.S, Toulouse, France); an Alpha Fox 4000 odor fingerprint analyzer (Alpha M.O.S, Toulouse, France); sensor models are shown in Table 4.

### 3.2. Chemical Composition and Sensory Characteristics

#### 3.2.1. Alcohol-Soluble Extract

Refer to the alcohol-soluble extract determination method (2201) in *Chinese Pharmacopoeia* (2020 edition) for testing.

A total of 3 g of CR powder, sieved according to Pharmacopoeia No. 2, was accurately weighed and placed in a conical flask. A total of 75 mL of 45% ethanol was added, and the flask was then weighed with a stopper in place. It was allowed to stand for 1 h before being refluxed and extracted for another hour. Upon completion, the mixture was cooled down again with the stopper in place, and an additional 45% ethanol was added to compensate for any weight loss. The mixture was thoroughly shaken and filtered. A total of 25 mL of filtrate was accurately measured and poured into a pre-weighted evaporating dish, which was then dried at 105 °C for 3 h. Following this, the dish was cooled in a dryer for 30 min. The dried product was promptly weighed, and the alcohol-soluble extract content (%) was calculated based on this weight.

#### 3.2.2. Polysaccharide

A quantity of 0.25 g of CR powder, sieved according to Pharmacopoeia No. 2, was accurately weighed and placed in a 50 mL conical flask. A total of 35 mL of 80% ethanol was added, and ultrasonic extraction was performed for 30 min. The filter residue was then cleaned with hot 80% ethanol. Subsequently, 35 mL of water was added to both the filter residue and filter paper, and ultrasonic extraction was performed again for another 30 min. Following this, water was added to filtrate and it was diluted to a 100 mL volumetric flask. A measure of 1 mL of this diluted filtrate was then transferred to a 5 mL volumetric flask to obtain the test solution.

Then, 200 μL of the test sample solution was accurately transferred into a 2 mL centrifuge tube, and 200 μL of freshly prepared 5% phenol solution was added, followed by 700 μL of concentrated sulfuric acid. The mixture was quickly blended. Subsequently, the color reaction was carried out in an 80 °C water bath for 10 min, and the reaction was terminated in an ice water bath for 5 min. Afterward, 100 μL of the reaction solution was accurately aspirated into a 96-well plate, and the absorbance values at 490 nm were measured using a microplate reader.

#### 3.2.3. Taste Characteristic

A liquid taste analyzer is equipped with seven sensors, among which the ANS sensor reflects sweetness. A measure of 1 g of CR powder, sieved according to Pharmacopoeia No. 2, was accurately weighed and placed in a 100 mL conical flask. Then, 50 mL of Watsons water was added, and the flask was weighed before being subjected to reflux extraction for 1 h. Following this, the mixture was cooled down and weighed again, and additional water was added to compensate for any weight loss. The mixture was then filtered while hot, and the filtrate was placed in a 50 mL centrifuge tube. The tube was centrifuged at 5000 r/min for 600 s, and the supernatant was collected and passed through a 0.45 μm microporous filter membrane. The filtered supernatant was then placed in a beaker specifically designed for use with an electronic tongue for detection.

During the detection process, the collection time was set to 120 s, and the sensors were cleaned with Watsons water for 10 s. Each sample was injected eight times, and the average of the stable sensor response values from the last 20 s of the last three injections was taken as the characteristic indicator of sweet taste.

#### 3.2.4. Color Characteristic

An appropriate amount of CR powder, sieved according to Pharmacopoeia No. 2, was weighed and placed in a culture dish equipped with a spectrophotometer. The dish featured a thickness of approximately 2~3 mm, a measuring aperture of 30 mm, an illumination light source of D65, and a standard observation angle of 10°. Each sample was measured three times, and b* values obtained were adopted as a characteristic indicator of yellowness.

### 3.3. E-Nose Analysis

An amount of 0.6 g of CR powder, sieved according to Pharmacopoeia No. 2, was accurately weighed and placed into a 20 mL headspace injection bottle. It was then incubated at 40 °C for 5 min. Subsequently, 2000 μL was injected, with the collection commencing for 120 s, followed by a delay of 600 s before collection. All other parameters were kept as default settings. Each sample was measured four times, and maximum response values from 18 sensors were adopted as the odor characteristic data for the sample.

### 3.4. Data Processing

A ChiPlot online mapping system (https://www.chiplot.online/, accessed on 5 October 2024) was used to draw a cloud and rain map, a radar map, and a correlation heat map. Origin 2021 was used to create an E-nose odor fingerprint; SPSS 20 software was used to carry out a Pearson correlation analysis and MLR, and MATLAB R2023b software was used to construct the RF, SVM, BP, and PSO-SVM regression prediction models.

## 4. Conclusions

Different samples of CR differed in chemical composition and appearance traits. CR has a special aroma, and prediction of alcohol-soluble extract content and polysaccharide extract, as well as prediction of taste and color could be achieved by odor information combined with SVM, which could reach an accuracy of more than 85%. To a certain extent, the prediction of the chemical composition and sensory information of CR based on the E-nose was realized. In order to improve the accuracy of models, we need more samples and further optimization of feature extraction as well as machine learning algorithms.

To assess the feasibility and accuracy of the detection method, and to procure authentic and reliable data, this study solely focused on regression prediction for two chemical fractions that possess established detection methods and are industry-recognized. These components were indirectly associated with odor profiles derived from the E-nose. However, in addition to alcohol-soluble extract content and polysaccharide extract—both of which are chemically complex fractions—CR also possesses other chemical constituents, such as lobetyolin, and its anti-oxidant, anti-inflammatory, and antitumor activities have been confirmed [24]. In addition, our group also investigated volatile compounds that were directly linked to the odor profile. Consequently, more chemical constituents will be tested, while volatile compounds will be included to further improve and enrich the prediction model. This will not only make the model more interpretable, but also facilitate an exploration of the correlations between these chemical constituents.

## Figures and Tables

**Table 1:** Prediction performance indicators of models.

Models	Training Set			Testing Set		
	RMSE	*R* ^2^	MAE	RMSE	*R* ^2^	MAE
RF	1.622	0.911	1.200	1.909	0.841	1.436
SVM	1.588	0.919	1.036	1.456	0.910	1.167
BPNN	1.538	0.926	1.046	1.577	0.879	1.369

**Table 2:** Forecast results.

Indicators	Training Set			Testing Set		
	RMSE	*R* ^2^	MAE	RMSE	*R* ^2^	MAE
alcohol-soluble extract	1.588	0.919	1.036	1.456	0.910	1.167
polysaccharide	3.076	0.918	1.984	3.654	0.859	3.026
sweetness values	21.167	0.793	12.824	22.527	0.807	16.854
yellow values	0.971	0.877	0.609	0.785	0.863	0.643

**Table 3:** Samples information.

Variety	Origin	1	2	3
*Codonopsis pilosula* (Franch.) Nannf.	Shanxi	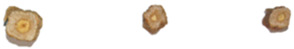		
	Gansu	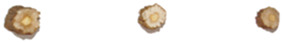		
*Codonopsis pilosula* Nannf. var. *modesta* (Nannf.) L.T.Shen	Gansu	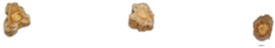		
	Sichuan	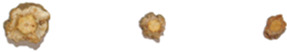		
*Codonopsis tangshen* Oliv.	Chonqing	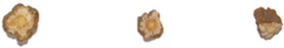		
	Hubei	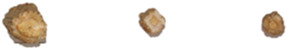		

**Table 4:** E-nose sensors models.

Number	Sensors Model	Detection Range
S1	LY2/LG	Gases with strong oxidizing ability
S2	LY2/G	Ammonia, organic amines, carbon oxides
S3	LY2/AA	Ammonia, ethanol, acetone
S4	LY2/GH	Ammonia, organic amines
S5	LY2/gCTL	Hydrogen sulfide
S6	LY2/gCT	Propane, butane
S7	T30/1	Organic compounds
S8	P10/1	Hydrocarbons
S9	P10/2	Methane, ethane
S10	P40/1	Gases with strong oxidizing ability
S11	T70/2	Aromatic compounds
S12	PA/2	Ethanol, ammonia, organic amines
S13	P30/1	Combustible gases, organic compounds
S14	P40/2	Gases with strong oxidizing ability
S15	P30/2	Ketones, hydrogen sulfide
S16	T40/2	Gases with strong oxidizing ability
S17	T40/1	Gases with strong oxidizing ability
S18	TA/2	Organic compounds

## Data Availability

The data presented in this study are available on request from the corresponding author, as the study is still ongoing.
